# Docking Prediction, Antifungal Activity, Anti-Biofilm Effects on *Candida* spp., and Toxicity against Human Cells of Cinnamaldehyde

**DOI:** 10.3390/molecules25245969

**Published:** 2020-12-16

**Authors:** Danielle da Nóbrega Alves, Alex France Messias Monteiro, Patrícia Néris Andrade, Josy Goldoni Lazarini, Gisely Maria Freire Abílio, Felipe Queiroga Sarmento Guerra, Marcus Tullius Scotti, Luciana Scotti, Pedro Luiz Rosalen, Ricardo Dias de Castro

**Affiliations:** 1 Graduate Program in Natural and Synthetic Bioactive Products (PgPNSB), Department of Clinic and Social Dentistry, Center for Health Sciences, Federal University of Paraiba, João Pessoa-PB 58051-900, Brazil; dnobregaalves@msn.com; 2 Graduate Program in Natural and Synthetic Bioactive Products (PgPNSB), Department of Pharmaceutical Sciences, Center for Health Sciences, Federal University of Paraíba, João Pessoa-PB 58051-900, Brazil; alexfrancem@gmail.com; 3 Experimental Pharmacology and Cell Culture Laboratory, Center for Health Sciences, Federal University of Paraiba, João Pessoa-PB 58051-900, Brazil; pattyneris@yahoo.com.br; 4 Department of Bioscience, Piracicaba Dental School, University of Campinas, Campinas-SP 13414-903, Brazil; josy662@hotmail.com (J.G.L.); rosalen@fop.unicamp.br (P.L.R.); 5 Department of Physiology and Pathology, Center for Health Sciences, Federal University of Paraíba, João Pessoa-PB 58051-900, Brazil; gisely_abilio@yahoo.com.br; 6 Department of Pharmaceutical Sciences, Center for Health Sciences, Federal University of Paraíba, João Pessoa-PB 58051-900, Brazil; felipeqsguerra@gmail.com; 7 Graduate Program in Natural and Synthetic Bioactive Products (PgPNSB), Department of Chemistry, Center for Health Sciences, Federal University of Paraíba, João Pessoa-PB 58051-900, Brazil; mtscotti@gmail.com; 8 Graduate Program in Natural and Synthetic Bioactive Products (PgPNSB), Cheminformatics Laboratory, Center for Health Sciences, Federal University of Paraíba, João Pessoa-PB 58051-900, Brazil; luciana.scotti@gmail.com; 9 Biological Sciences Graduate Program (PPGCB), Institute of Biomedical Sciences (ICB), Federal University of Alfenas (UNIFAL-MG), Alfenas 37130-000, Brazil; 10 Department of Clinic and Social Dentistry, Center for Health Sciences, Federal University of Paraiba, João Pessoa-PB 58051-900, Brazil

**Keywords:** candidiasis, antimicrobial products, antifungal, molecular coupling simulation

## Abstract

Objective: This study evaluated the antifungal activity of cinnamaldehyde on *Candida* spp. In vitro and in situ assays were carried out to test cinnamaldehyde for its anti-*Candida* effects, antibiofilm activity, effects on fungal micromorphology, antioxidant activity, and toxicity on keratinocytes and human erythrocytes. Statistical analysis was performed considering α = 5%. Results: The minimum inhibitory concentration (MIC) and minimum fungicidal concentration (MFC) of cinnamaldehyde ranged from 18.91 μM to 37.83 μM. MIC values did not change in the presence of 0.8 M sorbitol, whereas an 8-fold increase was observed in the presence of ergosterol, suggesting that cinnamaldehyde may act on the cell membrane, which was subsequently confirmed by docking analysis. The action of cinnamaldehyde likely includes binding to enzymes involved in the formation of the cytoplasmic membrane in yeast cells. Cinnamaldehyde-treated microcultures showed impaired cellular development, with an expression of rare pseudo-hyphae and absence of chlamydoconidia. Cinnamaldehyde reduced biofilm adherence by 64.52% to 33.75% (*p* < 0.0001) at low concentrations (378.3–151.3 µM). Cinnamaldehyde did not show antioxidant properties. Conclusions: Cinnamaldehyde showed fungicidal activity through a mechanism of action likely related to ergosterol complexation; it was non-cytotoxic to keratinocytes and human erythrocytes and showed no antioxidant activity.

## 1. Introduction

*Candida* genus is versatile and may colonize up to 70% of the population without any clinical signs of infection. However, local or systemic alterations can trigger an imbalance in the host and predispose them to candidiasis, which can range from a superficial and localized involvement to a mortal disease [1].

*Candida* infections are frequent in the oral cavity, especially in immunosuppressed individuals, users of dental prostheses, and with hyposalivation [2]. In addition to *C. albicans*, *C. krusei*, *C. tropicalis*, and *C. glabrata* are associated with oral candidiasis. These species have the abilities to form biofilms that can develop in oral mucosa, dental tissues, restorative materials, and denture prostheses [2,3].

In conventional therapy, the antifungal classes most often used are polyenes (nystatin and amphotericin B) and azoles (miconazole, clotrimazole, ketoconazole, itraconazole, and fluconazole). In some cases, however, it may be necessary to use voriconazole and posaconazole (triazoles), liposomal amphotericin B (liposome incorporated) or echinocandin [4]. In superficial infections, the most commonly used topical medications are nystatin, as an oral suspension, and miconazole. If topical therapy fails, systemic therapy with ketoconazole and fluconazole is indicated [5]. The major issue is that resistance to these drugs has increased due to their indiscriminate prophylactic use and self-medication [6].

Natural products with therapeutic properties have been considerably explored as an alternative to overcome microbial resistance and drug toxicity (cellular, tissue, neurological, renal, or hepatic) [7] observed in conventional antifungal therapy. Naturally occurring molecules that present significant efficacy against pathogenic fungi can be isolated and chemically engineered [8]. The use of products of natural origin, including essential oils and their constituents, has been associated with reduced costs, lower toxicity, and greater affordability when compared to prescription drugs. However, it is important to note that fungal resistance and failure to isolate the active principle(s) [9] may render alternative therapy less effective in terms of antimicrobial activity. In this scenario, long-term treatment may be needed for complete resolution.

Antifungal resistance has been reported for *C. albicans* upon long-term use of antifungal agents and recurrent mucocutaneous and oropharyngeal candidiasis. Other *Candida* spp., such as *C. krusei*, are intrinsically resistant and less susceptible to many classes of antifungal agents, most notably fluconazole. With the introduction of new classes of antifungal agents, reports of resistance have become increasingly common, particularly upon treatment with azoles (especially fluconazole) [10].

Mechanisms of resistance to polyene antifungals often occur due to overexpression and mutation in ERG1, ERG2, ERG3, ERG4, ERG6, ERG11 and ERG11 genes, which are involved in ergosterol biosynthesis. This leads to the accumulation of sterols other than ergosterol, resulting in dramatic disruption of cell membrane permeability [11,12,13]. For decades, polyenes were thought to provoke their toxic effects by intercalating into membranes containing ergosterol and forming channels that cross through the membrane, cause leakage of cellular components, and ultimately lead to cell death. Recently detailed structural and biophysical studies have demonstrated that polyenes bind to and extract ergosterol, the main fungal membrane sterol, thereby affecting essential cellular functions [14]. Resistance to echinocandins occurs due to mutations in FKS1 and FKS2 genes that encode the catalytic subunits of the enzyme 1.3-β-glucan synthetase, which is responsible for cell wall synthesis, the main target of echinocandins [11].

Cinnamaldehyde (cinnamaldehyde or 3-phenyl-2-propenal; C_9_H_8_O) is found naturally in the bark of *Cinnamomum zeylanicum* Blume and other Cinnamomum species such as camphor and cassia. It is a phenylpropanoid found in the form of a yellow oily liquid, with a cinnamon odor and sweet taste [15]. The literature presents many health benefits for *Cinnamomum zeylanicum* Blume, which include anti-Parkinson [16] and anti-Alzheimer activity [17]; anti-inflammatory properties [18,19], antimicrobial activity, preventive effects on cardiovascular disease and blood glucose control [19], platelet anti-aggregation and blood circulation [18] activities, anti-angiogenesis [20] and anti-HIV-1 properties [21].

The antifungal effects of cinnamaldehyde have been previously reported, with MIC values ranging from 378.3 μM to 30,266.6 μM [22,23]. Anti-biofilm action has also been reported in the literature [24,25]. However, the effect of cinnamaldehyde against *Candida* multispecies biofilm, a microbial organization often found in superficial infections, remains to be determined.

The mechanisms through which cinnamaldehyde acts against *Candida* spp. can be related to interference with membrane cell permeability, since it has been shown to reduce ergosterol biosynthesis [26]. Yet, the interaction of cinnamaldehyde molecules with ergosterol or enzymes involved in ergosterol biosynthesis or cell wall formation is largely unknown. Hence, in vitro and in silico studies determining molecular anchorage mechanisms of cinnamaldehyde may contribute to elucidate its molecular targets in yeast cells.

This study investigated the antifungal activity of cinnamaldehyde against different *Candida* spp., its potential mechanisms of action, molecular interactions with enzymes related to the yeast cell membrane, and wall formation. We further carried out molecular docking analysis and tested cinnamaldehyde for its effects on biofilm adherence and micromorphology, as well as antioxidant activity and cytotoxicity in keratinocytes and human erythrocytes.

This investigation contributes to improving the knowledge about the biological activity of cinnamaldehyde as it presents the following innovative data: extensive anchorage molecular analysis (docking and redocking assays), the anti-biofilm effect on *Candida* multispecies, effects of this molecule on fungal micromorphology, and its toxicity against human erythrocytes and keratinocyte cells. The results found indicate that the cinnamaldehyde is promising in futures investigations, as in vivo and clinical evaluations to determine the safety and antifungal effect.

## 2. Results

### 2.1. Molecular Dockings

As demonstrated in Table 1, cinnamaldehyde presented negative ligand-receptor interaction energy at the molecular targets, demonstrating affinity mainly for squalene epoxidase (−70.4951 kcal mol^−1^) and thymidylate synthase (−66.4852 kcal mol^−1^).

**Figure 1:**
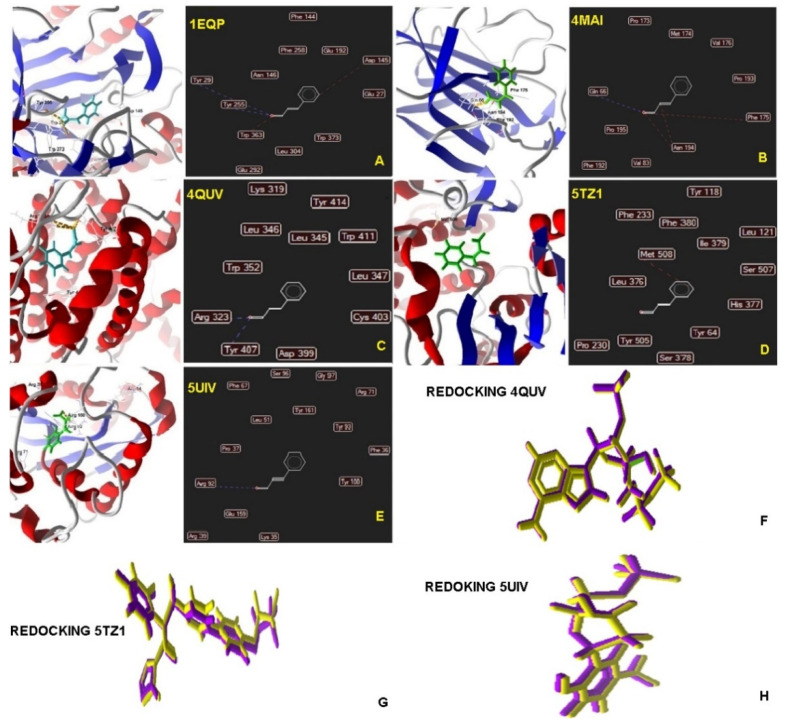
Map of interactions between the PDB ID protein and cinnamaldehyde. (**A**) Map of interactions between the PDB ID 1EQP protein (1,3-β-glucan synthase) and cinnamaldehyde; (**B**) Map of interactions between the PDB ID 4MAI protein (squalene epoxidase) and cinnamaldehyde; (**C**) Map of interactions between the PDB ID 4QUV protein (Δ-14-sterol reductase) and cinnamaldehyde; (**D**) Map of interactions between the PDB protein ID 5TZ1 (14-α-demethylase) and cinnamaldehyde; (**E**) Map of interactions between the PDB ID 5UIV protein (thymidylate synthase) and cinnamaldehyde; (**F**) Map of interactions with the protein PDB ID 4QUV (Δ-14-sterol reductase)—redocking: overlapping the binder with the binder pose in redocking; (**G**) Map of interactions with PDB ID protein 5TZ1 (14-α-demethylase)—redocking: overlapping the binder with the binder pose in redocking; (**H**) Map of interactions with protein PDB ID 5UIV (thymidylate synthase)—redocking: overlapping of the binder with the binder pose in redocking.

As shown in Figure 1A, the interaction between 1EQP (1,3-β-glucan synthase), cinnamaldehyde and the standard drugs are mediated by hydrogen bonds (depicted in blue) with amino acid residues (cinnamaldehyde: Tyr29, Tyr255; miconazole: Asn146, Asn191; nystatin: Tyr317). The structures in blue, red, and green correspond to hydrogen, steric and electrostatic bonds, respectively.

Hydrogen bonds (blue) were detected between the 4MAI protein (squalene epoxidase) and the three compounds (cinnamaldehyde, nystatin, and miconazole) with specific amino acid residues (cinnamaldehyde: Gln66; miconazole: Trp81; nystatin: Ala177, Val188, Arg137, Thr184, Asn178, Asn180, Cys182). In addition, steric (red) interactions were observed at Phe175 residue (in common with cinnamaldehyde and nystatin), and at Lys77 (in common with miconazole and nystatin), as shown in Figure 1B.

Hydrogen bonds (blue) were detected between the inhibitor [NADPH Dihydro-Nicotinamide-Adenine-Dinucleotide Phosphate (NDP)] complexed with the 4QUV protein (Δ-14-sterol reductase) and the three compounds (cinnamaldehyde, nystatin, and miconazole), with common amino acid residues (cinnamaldehyde, miconazole, and the ligand: Tyr407; miconazole and ligand: Leu347; nystatin and ligand: Thr254; nystatin, miconazole, cinnamaldehyde, and the ligand: Arg323). In addition, steric bonds (red) were observed at Arg323 residue (in common with cinnamaldehyde, nystatin, miconazole, and the ligand) and at Trp411, Cys403 and Lys319 (in common with miconazole and nystatin), as depicted in Figure 1C.

Hydrogen bonds (blue) were also detected between the inhibitor [(*R*)-2-(2,4-Difluorophenyl)-1,1-difluoro-3-(1*H*-tetrazol−1-yl)−1-(5-(4-(2,2,2-trifluoroethoxy)phenyl)pyridin−2-yl)propan-2-ol (VT1)] complexed with the 5TZ1 (14-α-demethylase) protein and the three compounds (cinnamaldehyde, nystatin, and miconazole), with amino acid residues in common with nystatin, such as Met508, Tyr118, Gly307, Gly303, Met306, His377, and Gly308. In addition, steric (red) interactions in common with miconazole and the binder were detected at Gly307 residue. Cinnamaldehyde, miconazole, and the binder presented interactions at Met508 residue, as shown in Figure 1D.

Hydrogen bonds were detected between the inhibitor [Thymidine-5′-Phosphate (TMP)] complexed with the 5UIV (thymidylate synthase) protein and the three compounds (cinnamaldehyde, nystatin, and miconazole), with common amino acid residues (cinnamaldehyde, miconazole, and binder: Arg92; miconazole and binder: Lys35; nystatin, miconazole, and binder: Arg39). Common steric (red) interactions between cinnamaldehyde, nystatin, miconazole, and the binder were detected at Arg323 residue, and between miconazole and nystatin at Trp411, Cys403, and Lys319, as shown in Figure 1E.

Redocking analysis was performed to validate the molecular docking based on the overlap deviation of two identical protein structures, each using the same anchoring configuration of the test compounds. Table 1 shows the RMSD of proteins that presented ligands, namely: PDB ID 4QUV (Δ-14-sterol reductase): 0.2048; PDB ID 5TZ1 (14-α-demethylase): 0.4409; PDB ID 5UIV (thymidylate synthase): 0.2112. Mean square standard deviation values less than 1.0 indicate greater reliability of the docking data, as lower RMSD values translate into more reliable outcomes.

Figure 1F shows the redocking data of 4QUV (Δ-14-sterol reductase) protein binder. The overlapping of the binder with its pose yielded a deviation value (RMSD) of 0.2048; see Table 1. The common residues for redocking through hydrogen bonds were: redocking and binder—Tyr414, Lys406, Tyr407, Asp399, Leu347; redocking and cinnamaldehyde—Tyr407; redocking and nystatin—Lys319, redocking and miconazole—Leu347 and Tyr407; redocking and cinnamaldehyde, nystatin and miconazole—Arg323. The steric bond (red) residues in common were: redocking, binder, nystatin and, miconazole—Arg323. The electrostatic (green) interactions in common were redocking and binder, miconazole, and nystatin—Arg323.

Figure 1G depicts the redocking data for the 5TZ1 (14-α-demethylase) ligand. As seen in Table 1, the overlap of the binder with its pose has a standard deviation value (RMSD) of 0.4409. The common residues presenting steric bonds (red) were: redocking, binder, cinnamaldehyde, and miconazole—Met508; redocking and binder—His377; redocking and miconazole—Leu376.

Figure 1H depicts the redocking data for the 5UIV ligand protein (thymidylate synthase). According to Table 1, the overlap of the binder with its pose has a deviation value (RMSD) of 0.2112. The residues that presented hydrogen bonds (blue) in common were: redocking, binder, cinnamaldehyde, and miconazole—Arg92; redocking, binder, nystatin and miconazole—Arg39; redocking, binder and miconazole—Lys35, redocking and binder—Arg71. The common residues with steric bonds (red) were: redocking, binder, nystatin—Gly97, Lys35; redocking and binder—Tyr100, Phe67. The common residues with electrostatic interactions were: redocking and binder—Arg92, Lys35.

### 2.2. Determination of the Minimum Inhibitory Concentration and Minimum Fungicidal Concentration

The antifungal effects of cinnamaldehyde on the growth of different yeast strains were tested. The minimum inhibitory concentration (MIC) and minimum fungicidal concentration (MFC) values for cinnamaldehyde and nystatin are shown in Table 2. The MIC and MFC values of cinnamaldehyde ranged from 18.91 to 37.83 μM, whereas the MIC and MFC values of nystatin ranged from 8.55 to 34.67 μM. The carrier used for emulsion (distilled water and Tween 80) did not affect yeast growth. The MFC/MIC ratio indicated that cinnamaldehyde presents fungicidal effects against all tested strains.

### 2.3. Sorbitol and Ergosterol Assays

Sorbitol is an osmotic protector that minimizes the effect of chemical agents on the fungal cell wall. In our study, the presence of an osmotic protector did not affect the minimum concentration of cinnamaldehyde required to inhibit cell growth of either tested strains (*C. albicans* ATCC 90028 and *C. tropicalis* CBS 94), suggesting that its mechanism of action is not likely related to disruption of the cell wall (Table 3). In contrast, the control drug caspofungin had its MIC increased (from 0.057 to 0.114 μM) in the presence of sorbitol as it has a known effect on cell wall biosynthesis.

Next, cinnamaldehyde was tested for its potential effects on the yeast cell membrane. Our findings revealed that in both tested strains, the MIC of cinnamaldehyde increased (from 37.8 μM to >302.6 μM) in the presence of exogenous ergosterol, which suggests a likely disruption of cell membrane permeability (Table 4).

### 2.4. Effects of Cinnamaldehyde on Fungal Micromorphology

**Figure 2:**
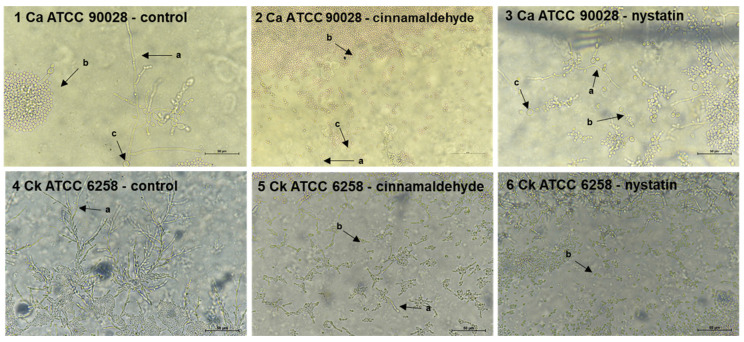
The effects of cinnamaldehyde and nystatin on the micromorphology of *C. albicans* ATCC 90028 and *C. krusei* ATCC 6258. (**1** and **4**) yeast control; (**2** and **5**) treatment with cinnamaldehyde at 37. 83 μM; (**3** and **6**) treatment with nystatin at 81.43 μM. a: pseudohyphae. b: blastoconidia. c: chlamydoconidia. Bar: 50 μm (400×).

Untreated *C. albicans* ATCC 90028 and *C. krusei* ATCC 6258 microculture showed the presence of pseudo-hyphae, blastoconidia, and chlamydospores. In contrast, treatment with cinnamaldehyde (37.83 μM) or nystatin (81.43 μM) promoted changes in the filamentous form. Cinnamaldehyde-treated cells showed impaired cellular development, with an expression of rare pseudo-hyphae and absence of chlamydoconidia. These results are present in Figure 2.

### 2.5. Antibiofilm Activity of Cinnamaldehyde

**Figure 3:**
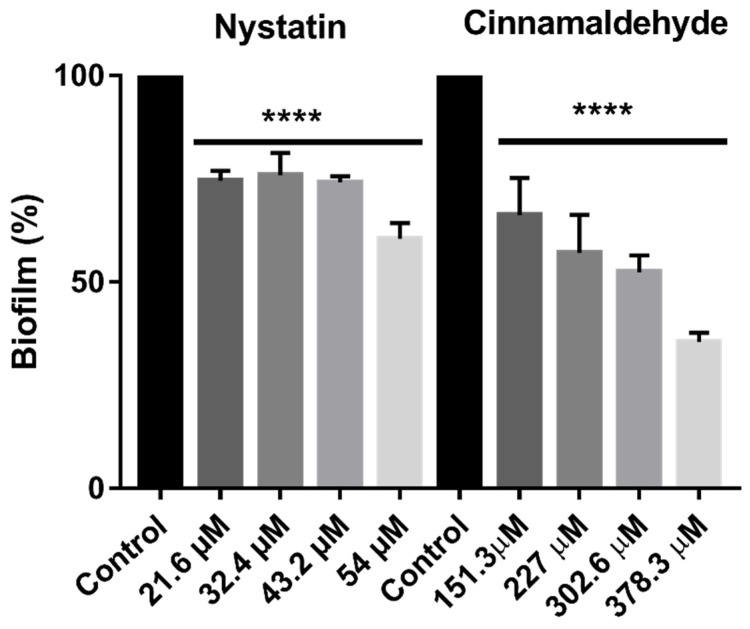
Inhibitory effect (mean, standard deviation) of cinnamaldehyde and nystatin against multi-species *Candida* biofilm. Results presented as mean ± SD of three independent experiments performed in triplicate (One-way ANOVA with Tukey’s post-test, **** *p* < 0.0001).

Cinnamaldehyde reduced biofilm by 64.52% to 33.75% (*p* < 0.0001) at low concentrations (378.3—151.3 µM). Nystatin, used as a control, reduced biofilm adherence by 39.54 to 25.44%) (*p* < 0.0001). The vehicle did not affect biofilm adherence. Figure 3 shows the effect of cinnamaldehyde on reduced *Candida* multispecies biofilm adherence.

### 2.6. Cytotoxicity of Cinnamaldehyde in Keratinocytes

**Figure 4:**
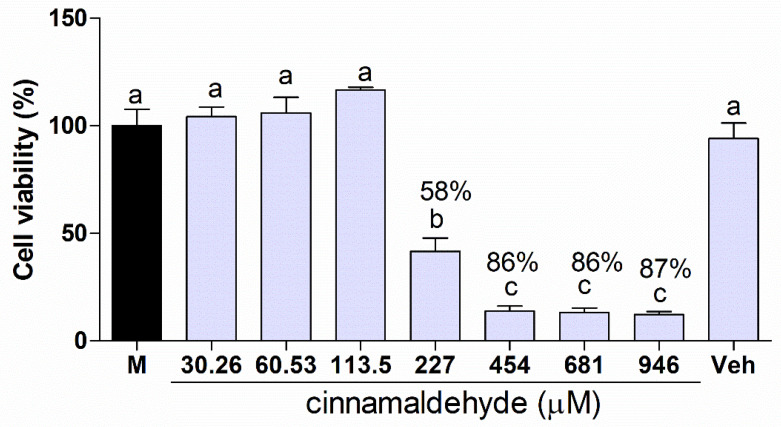
Viability of Human keratinocyte cells (HaCaT) treated with control culture medium (M); different concentrations of cinnamaldehyde and vehicle (DMSO, 946 µM) (Veh). All groups were compared with the control. Different symbols indicate statistically significant differences. The symbol % indicates reduced cell viability. Results presented as mean ± SD of three independent experiments performed in triplicate. a, b, c—Different letters indicate statistical differences (One-way ANOVA followed by Tukey’s post-hoc test, *p* < 0.05).

As seen in Figure 4, cinnamaldehyde at 30.26 µM, 60.53 µM, and 113.5 µM did not exert toxic effects on HaCaT cells compared to the control (*p* > 0.05). However, cinnamaldehyde decreased cell viability at concentrations higher than 227 µM (*p* < 0.05). In addition, the vehicle (DMSO) at 946 µM did not have cell toxicity compared to the control culture media (M) (*p* > 0.05).

### 2.7. Cytotoxicity in Human Erythrocytes

**Figure 5:**
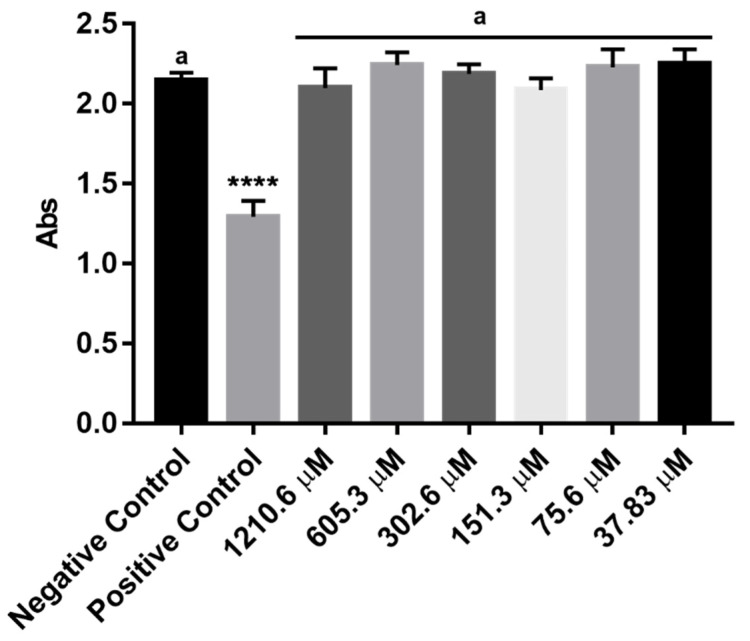
The cytotoxic effects of cinnamaldehyde against human erythrocytes. Blood Type A+, B+, AB+, and O+ Human Erythrocytes were exposed to different concentrations of cinnamaldehyde, and hemolytic activity was measured by colorimetric assay. Results presented as mean ± SD of three independent experiments performed in triplicate. One-way ANOVA followed by Tukey’s post-test was performed for comparison between groups, **** *p* < 0.0001 relative to the negative control. Letter a indicates no significant statistical difference between groups.

Cinnamaldehyde was further tested for its effects against human erythrocytes. As shown in Figure 5, cinnamaldehyde did not promote significant alterations in cell viability at different concentrations (37.83 µM–1210.6 µM) when compared to the negative control, resulting in hemolysis of red cells HC_50_ value > 1210.6 µM.

### 2.8. Antioxidant Activity of Cinnamaldehyde by the DPPH Method

Cinnamaldehyde and Trolox (control) were tested for their antioxidant activity by the DPPH method and showed IC_50_ values of 1.74 mM and 1.88 mM, respectively. These results are presented in Table 5.

## 3. Discussion

Molecular docking analysis serves to predict molecular interactions (e.g., electrostatic interactions, Van der Waals, Coulombic, and hydrogen bonds) between two structures, most commonly a receptor protein and a ligand. Docking analysis calculates the energy values necessary to perform the coupling in each of the possible linking sites. While the simulation is running the energy of the currently best-found pose (the pose with the lowest energy) can be observed and it was selected (Table 1). Lower energy values translate into stronger binding sites [27].

A comparative analysis between cinnamaldehyde and gold-standard drugs for the treatment of superficial candidiasis (nystatin and miconazole) was carried out [28]. Energy binding values of molecular targets in the yeast cell and a preview of possible molecular interactions and efficacy were obtained.

In this study, cinnamaldehyde presented negative ligand-receptor interaction energy at the molecular targets, demonstrating affinity mainly for squalene epoxidase and thymidylate synthase. Similar results were observed for miconazole, which showed affinity for most of the targets, except squalene epoxidase. In contrast, nystatin presented affinity only for 1,3-β-glucan synthase. Nystatin and miconazole are known for their effectiveness in treating superficial infections in different body sites [29].

The proteins involved in the constitution of the fungal cell wall and membrane, as well as the enzymes that are involved in its biosynthesis, are excellent targets for antifungal chemotherapy [30,31]. The results obtained in the molecular docking analysis support the hypothesis that cinnamaldehyde affects *Candida* spp., including inhibition of biofilm formation, once that molecular targets involved with important cellular functions (1,3 β-glucan synthase, squalene epoxidase, δ-14-sterol reductase, 14-α-demethylase, and thymidylate synthase) may be affected by this molecule.

The yeast cell wall consists of a covalently branched 1,3-β-glucan linked with a chitin interchain. This structure contributes to the formation of biofilms that can be recognized by the host’s immune system [30]. Thymidylate is an enzyme involved in the synthesis of 2′deoxythymidine-5′-monophosphate (dTMP), a nucleotide that makes up deoxyribonucleic acid (DNA) [32]. In addition to the targets present in the fungal cell and nucleus, the sterols present in the plasma membrane are essential for maintaining its structure and functions. The complex biosynthesis of ergosterol involves almost 30 enzymes known as Erg proteins, among which are squalene epoxidase, δ-14-sterol reductase, and 14-α-demethylase—which were evaluated in our study [31].

The results obtained in the molecular docking analysis point to a possible affinity of all the analyzed proteins to the test site, where the use of different targets increases the possibility of success in proposing antifungal activity, corroborated by the biological test of cinnamaldehyde against *Candida* spp.

In the literature, different MIC values against yeast strains have been reported for cinnamaldehyde. A study reported MIC of 378.3 μM on *C. albicans* ATCC 7965 [22], while other authors found MIC of 3026.6 μM and 3783.2 μM against clinical isolates of *C. albicans* and *C. tropicalis* [23]. Clinical isolates generally show an increase in resistance to antimicrobials due to frequent exposure to them and consequent genetic mutations. Another study tested cinnamaldehyde against *Candida* and reported MIC of 945.8 μM on *C. albicans* ATCC 10231 and 90028 [33]. The differences in these results can be explained by type of technique used, strain resistance, effectiveness of cinnamaldehyde isolation process, and the type of strain used (reference or clinical isolate). Besides that, this study used the protocol proposed by the Clinical and Laboratory Standards Institute [34], which recommends a fungal cell density different from that of the previous studies with *C. albicans* ATCC 90028. A study compared the micromorphology of reference and clinical strains, showing that treated cultures had fewer structures (e.g., pseudo-hyphae and blastoconidia), which may be an indicative of the sensitivity of *C. albicans* ATCC 76645 to nystatin [35].

These promising results point to a new supply of antifungal agents, especially considering the observed MIC values and fungicidal character presented against all tested strains. Nystatin is considered the standard drug for the treatment of superficial candidiasis [36]. A study showed that cinnamaldehyde is effective against several clinical isolates resistant to fluconazole, with dose-dependent reduction of ergosterol content in the cell membrane [37].

The molecular targets of cinnamaldehyde in *Candida* spp. are still not well established and require further research addressing microbial response [38]. Evidence currently available suggests that cinnamaldehyde may affect the cell membrane [39].

Some studies confirm that cinnamaldehyde interacts in the membranes of microbial cells, increasing permeability, and thus disintegration of the cellular envelope [40,41,42]. The use of cinnamaldehyde has also been shown to be associated with changes in cell morphology, as well as damages to the wall and plasma membrane [37].

The ability of *Candida* species to produce filamentous structures, such as pseudohyphae or hyphae, is closely related to fungal virulence. The development of this structure increases the ability of fungal cells to invade host tissues [43]. The reduction in pseudohyphae extension in both *C. albicans* and *C. krusei* by cinnamaldehyde highlights its potent anti-virulence effects, which is in line with previous reports [25,44].

Biofilm adherence contributes significantly to drug resistance and is considered an important virulence factor [45,46], posing a need for alternative therapeutic approaches. This is the first study reporting the effects of cinnamaldehyde against multi-species *Candida* biofilm adherence

Cinnamaldehyde concentrations capable of inhibiting *Candida* multispecies biofilm showed a cytotoxic effect on human erythrocytes. However, these concentrations do not appear to exert a toxic effect on keratinocytes, indicating that the molecule under study has potential for topical use in keratinized mucosa. These results suggest conducting studies for topical toxicological evaluation in animals.

Cinnamaldehyde was tested for its cytotoxic effects in HaCaT cells, which are an important component of the oral epithelium [47]. Due to the insolubility of the compound in aqueous media, it was dispersed in Tween 80 (Sigma-Aldrich, St. Louis, MO, USA), as described in the materials and methods section. The cytotoxicity of free compounds would have been reduced in the absence of Tween 80 because of its non-polar character [48]. Cinnamaldehyde did not affect cell viability at concentrations up to 227 µM. At this concentration, HaCat viability was reduced to under 70%, which is the lowest value established by ISO 10993−5 for a given agent to be considered non-cytotoxic [49]. Previous studies have suggested that treatment with cinnamaldehyde could reduce cell viability to approximately 50% at concentrations ranging from 75 μM (adenocarcinoma cell line) to 226.9 μM (HaCaT keratinocytes) [48,50]. In our study, subtoxic concentrations ranged from 30.26 μM to 113.4 μM. However, the MIC values of cinnamaldehyde varied between 18.9 μM and 37.8 μM. These MIC values are up to three-fold lower than the highest subtoxic concentrations, which suggests that cinnamaldehyde could be safe for keratinocytes in the event of antifungal treatment.

The present study demonstrates the fungicidal effects and promising mechanisms of cinnamaldehyde for use as an antifungal. The results point to potential treatment of *Candida* infections. Further tests should be carried out, such as toxicological studies, as well as in vivo studies and clinical trials, which are necessary to establish the therapeutic efficacy and safety of cinnamaldehyde as an antifungal agent. Considering that cinnamaldehyde presents predicted mechanisms of reaction with nucleophiles (concomitant Schiff base formation), it is important to determine the potential to promote skin and mucosal sensibilization using animal models [51].

The toxicological analysis in human erythrocytes indicates possible damages promoted by molecules to the cell membrane, a structure responsible for the maintenance of cellular functions like passive and active transport, and production of ionic and electric gradients [52].

The study of new antifungal molecules should consider possible toxic effects on human cells. This in vitro assay for hemolytic activity is considered an alternative and preliminary method to screen bioactive molecules for their cytotoxicity. This rapid and highly reproducible test indicates the possible damages to the erythrocyte cell membrane produced by a given agent [53,54].

In our study, treatment with cinnamaldehyde at concentrations effective against planktonic (18.91 μM to 37.83 μM) and multispecies *Candida* biofilm cells (18.91 μM to 37.83 μM) did not cause cytotoxic effects on human erythrocytes.

Essential oils, in general, have antioxidant activity, and this action can contribute to delay cellular lipid peroxidation. We have improved the discussion on antioxidant activity. As observed in a previous study [55], cinnamaldehyde did not show antioxidant activity. This may be due to the low solubility of the compound under the conditions proposed by the method. The evaluation of this biological property contributes to the expansion of knowledge about cinnamaldehyde, thus contributing to the formulation of new research hypotheses related to the proposal of a pharmaceutical formulation for clinical use.

The discovery of molecules with antifungal potential can represent a new alternative for the treatment of biofilm infections. Our molecular anchorage analysis indicated different possibilities of interaction of cinnamaldehyde with receptor proteins on the fungal cell. Further studies should test the effects of cinnamaldehyde against resistant strains. In addition, the organic synthesis and chemical engineering of cinnamaldehyde analogs may yield a novel compound with promising biological activity.

## 4. Materials and Methods

### 4.1. Molecular Docking

The chemical structures of cinnamaldehyde and those of the standard drugs (nystatin and miconazole) were downloaded from the PubChem chemical structures database (https://pubchem.ncbi.nlm.nih.gov). The chemical structures were then imported into the HyperChemTM program (Hipercube, Gainesville, FL, USA) (RMS 0.1 kcal mol^−1^ in 600 cycles) [56] for energy minimization using molecular (MM+) and semi-empirical (AM1) methods [57,58]. The molecules were grouped into a single SDF file created by ChemAxon © Standardizer18.21.0 software (ChemAxon, Boston, MA, USA), containing the optimized structural data, to serve as input for molecular docking [59,60,61].

The investigated receptors (PDB IDs) were: 14-α-demethylase (PDB ID: 5TZ1) [62] whose binder is (*R*)-2-(2,4-Difluorophenyl)-1,1-difluoro-3-(1Htetrazol-1-yl)-1-(5-(4-2,2,2trifluoroethoxy)phenyl)pyridin-2-yl)propan-2-ol (VT1); Δ-14-sterol reductase (PDB ID: 4QUV) [63], which binds to NADPH Dihydro-Nicotinamide-Adenine-Dinucleotide Phosphate (NDP); 1,3-β-glucan synthase (PDB ID: 1EQP) [64]; and thymidylate synthase (PDB ID 5UIV) [65], whose binders are Thymidine-5′-Phosphate (TMP) and squalene epoxidase (PDB ID 4MAI) [66]. Receptor selection was defined according to current medication targets recommended for the treatment of candidiasis. The enzymes were downloaded from the Protein Data Bank (http://www.rcsb.org/pdb/home/home.do) (PDB format), and their binders and receptors were subjected to molecular docking using Molegro Virtual Docker 6.0 (MVD) (Molexus, Rört, Denmark) [67,68,69]. The Number of runs specifies is the number of times that the docking simulation is repeated for each ligand chosen to be docked. Sometimes more than one run is needed to identify promising poses (in particular for ligands having more than 15 flexible torsions or if no promising cavities exist). A discrete grid with a resolution of 0.8 Å covering the protein is created. At every grid point a sphere of radius 1.4 Å is placed. It is checked whether this sphere will overlap with any of the spheres determined by the Van der Waals radii of the protein atoms. Grid points where the probe clashes with the protein atom spheres will be referred to as part of the inaccessible volume, all other points are referred to as accessible.

The results are presented as total energy values for the Moldock Score ligand-receptor interaction. For proteins bearing ligands, redocking of the complexed ligands together with the respective proteins was carried out using Root Mean Square Deviation (RMSD) values [70,71,72,73].

The MolDock Score was used to perform the molecular docking calculations, considering the following parameters: intern energy, hydrogen binds and Sp2-Sp2 torsions, bind site X: −20.96, Y: −8.94, Z: 26.76 and 15 of radius, Moldock SE algorithm, 10 executions, up to 1500 interactions, with maximum population size equal to 50, with a maximum of 5 poses returned, and simplex evolution and standard configurations for the generation poses.

The RMSD indicates the modeling reliability, in that lower values are more favorable. This parameter is calculated based on the deviation of the overlapping of the ligand structures complexed with the protein and the pose generated from the molecular docking of that same ligand, using the same modeling configurations, as previously described. For the proteins with ligands complexed together with these structures, templates were created using these ligands as a reference, whereas the cavities that best presented molecular docking results were detected for the other proteins.

### 4.2. Chemicals and Microorganisms

Reference *Candida* spp. strains were obtained from the American Type Culture Collection (ATCC, Rockville, MD, USA) and the Central Bureau voor Schimmelcultures (CBS): *C. albicans* CBS 562, *C. albicans* ATCC 90028, *C. krusei* CBS 573, *C. krusei* ATCC 6258, *C. tropicalis* CBS 94, and *C. glabrata* ATCC 90030. Nystatin, tween 80%, trolox, cinnamaldehyde, and ergosterol were obtained from Sigma-Aldrich^®^ Chemical Co. (St. Louis, MO, USA), and sorbitol (d-sorbitol anhydrous) from INLAB^®^ (São Paulo, Brazil).

The following drugs and reagents used for cell viability assays were obtained from Sigma-Aldrich^®^ Chemical Co. (St. Louis, MO, USA): L-glutamine, penicillin, streptomycin sulfate, RPMI 1640 medium, MTT 3-(4,5-dimethylthiazol-2-yl)—RMSD indicates the modeling reliability in this study, lower values being favorable. This parameter is calculated based on the deviation of the overlapping of the structures of the ligand complexed with the protein and the pose generated from the molecular docking of that same ligand, using the same modeling configurations already mentioned.

For proteins with ligands complexed together with these structures, templates were created using these ligands as a reference, the others have detected the cavities that best presented results of molecular docking. 2,5-diphenyltetrazolium bromide). Keratinocytes (HaCaT strain) were obtained from the Rio de Janeiro cell bank (ATCC, Rio de Janeiro, Brazil). Fetal bovine serum (FBS) was obtained from Gibco (Grand Island, NY, USA). Ethanol was purchased from Merck^®^ (Darmstadt, Germany).

### 4.3. Determination of the Minimum Inhibitory Concentration (MIC) and Minimum Fungicidal Concentration (MFC)

The MIC was determined through the microdilution technique described by the Clinical and Laboratory Standards Institute [34]. Yeast suspensions were prepared in RPMI broth (Roswell Park Memorial Institute) media and adjusted by turbidity equivalence to 2.5 × 10^3^ CFU/mL, at 530 nm, abs 0.08–0.1 [34].

Serial dilutions of the test substances were made in 96-well U-bottom microtiter plates containing sterile RPMI, in triplicate. The plates were incubated for 24 h at 35 °C, and the results were read by visual observation of cell aggregates at the bottom of the wells [34]. Cinnamaldehyde, whose molecular weight is 132.16 g/mol, was tested at concentrations ranging from 302.6 to 2.364μM. Nystatin (Sigma-Aldrich, São Paulo, SP, Brazil) was used as a positive control and tested at concentrations ranging from 129.57 to 1.01μM. Strain viability and media sterility controls were included simultaneously in the assay; DMSO (dimethyl sulfoxide) (Sigma-Aldrich, São Paulo, Brazil) was used for the preparation of nystatin solution, and tween 80 (Sigma-Aldrich) for the preparation of the cinnamaldehyde solution.

The MFC was defined as the lowest concentration of the drug able to inhibit visible growth on solid media [74]. Aliquots from the wells corresponding to the MIC and higher concentrations were subcultured onto Sabouraud Dextrose agar (KASVI1, Kasv Imp and Dist. Prod/Laboratórios LTDA, Curitiba, Brazil). The plates were incubated for 24 h at 35 °C, and reading was performed by visual observation of fungal growth on the solid media based on the counting of Colony-Forming Units (CFU). The MFC/MIC ratio was calculated to determine whether the substance had fungistatic (MFC/MIC ≥ 4) or fungicidal (MFC/MIC < 4) activity [75].

### 4.4. Effects of Cinnamaldehyde on the Fungal Cell Wall and Membrane Permeability

#### 4.4.1. Sorbitol Test (Effect on Cell Wall)

For this assay, the MIC value was defined as the lowest concentration of the substance inhibiting visible microbial growth in the presence of sorbitol (D-sorbitol anhydrous) (INLAB, São Paulo, Brazil) [76,77]. The microdilution technique was used to compare the MIC values of cinnamaldehyde against *C. albicans* strains ATCC 90028 and *C. tropicalis* CBS 94 in the absence and presence of sorbitol at 4.39 μM. The technique was performed following the same procedures described in Section 4.3. The plates were incubated at 35 °C, and readings were performed 24 h after incubation [78,79]. The positive control for this assay was caspofungin at an initial concentration of 21.95 μM (caspofungin diacetate—Sigma-Aldrich, St. Louis, MO, USA), which is known to disrupt the yeast cell wall [80,81,82].

Based on the ability of sorbitol to act as an osmotic protector in the fungal cell wall, higher MIC values in the presence of sorbitol (standard medium) suggest that the cell wall is a likely cellular target of the compound under analysis.

#### 4.4.2. Ergosterol Test (Effect on Cell Membrane)

For this assay, the MIC was defined as the lowest concentration of the substance inhibiting visible microbial growth in the presence of exogenous ergosterol. The assay was performed using the microdilution technique, as previously described, in the presence of exogenous ergosterol (Sigma-Aldrich, São Paulo, Brazil) at a concentration of 1008.44 μM. The strains used in this test were the same as those described in Section 4.3. The plates were incubated at 35 °C, and the readings were performed after 24 h [78,79]. Nystatin was used as a positive control at the concentration of 129.57 μM for its known activity on yeast cell membranes, binding to membrane sterols and thereby disrupting membrane permeability [83]. A control with 96% ethanol and tween 80 (used to prepare ergosterol solutions) was also included.

### 4.5. Effects of Cinnamaldehyde on Fungal Micromorphology

The effects of cinnamaldehyde against pseudohyphae, chlamydospores, and blastoconia of *C. albicans* ATCC 90028 and *C. krusei* ATCC 6258 were assayed by microculture assay using melted rice agar (CA, HiMedia Laboratories, Mumbia, India) plus Tween 80 [43,84]. Cinnamaldehyde was added to the culture media before solidification at a concentration corresponding to the MIC. Nystatin was used as a control. Yeasts were seeded on glass slides, and the plates were incubated at 35 °C for 48 h. The slides were examined under light microscopy (Nikon Eclipse Ci^®^, Tokyo, Japan) at 400× magnification to determine the formation or absence of characteristic structures of *C. krusei and C. albicans* strains.

### 4.6. Effects of Cinnamaldehyde on Biofilm Reduction

The effects of different concentrations of cinnamaldehyde against mixed-species biofilm reduction (*C. albicans* ATCC 90028, *C. albicans* CBS 562, *C. krusei* CBS 573, and *C. tropicalis* CBS 94) were determined according to a microdilution protocol adapted from [78]. Briefly, 100 µL of Sabouraud Dextrose Broth (SDB, KASVI, Curitiba, Brazil) were added to 96-well U-bottom microdilution plates, then 100 µL of cinnamaldehyde solution (ranging from 378.3—151.3 µM) were added to the wells. Lastly, 100 µL of yeast inoculum (25 µL of each strain at 2.5 × 10^6^ CFU/mL) prepared with Sabouraud Broth plus sucrose (2%) were added to the wells. Nystatin (Sigma-Aldrich, São Paulo, Brazil) was used as a control. Media sterility and untreated growth controls were also included in all assays. The plates were incubated at 35 °C for 48 h. Biofilm was quantitated using 0.4% crystal violet (*w/v*), followed by dissolution in 95% ethanol. The optical density of 95% ethanol was measured at 595 nm (Multiskan GO; Thermo Fisher Scientific). Inhibition of adherence was measured indirectly considering the yeast growth group as 100% of fungal adherence [85].

### 4.7. MTT Cell Viability Assay

Human keratinocyte (HaCaT) cell cultures were obtained from the Bank of Cells of Rio de Janeiro (Rio de Janeiro, Brazil, code nh-skp-KT0046) and cultured in Dulbecco’s medium (DMEM) supplemented with 10% fetal bovine serum plus 100 U/mL penicillin, 100 μg/mL streptomycin sulfate and L-glutamine (37 °C, 5%, CO2). To determine the effects of cinnamaldehyde on cell viability, HaCaT cells were cultured in 96-well plates (1 × 106 cells/well) and incubated overnight. The cells were treated with cinnamaldehyde at 30.26; 60.53; 113.5; 227; 454; 681 and 946 µM for 24 h. The negative control group received only the vehicle (0.9% saline). MTT solution (0.3 mg/mL) was added to each well (200 μL), and the plates were incubated for an additional 3 h under the same conditions. The supernatant was removed and 200 μL of ethanol was added to the wells to lyse the cells. Absorbance was measured at 470 nm using an ELISA microplate reader [86].

### 4.8. Cytotoxic Effects of Cinnamaldehyde on Human Erythrocytes

The hemolytic activity of cinnamaldehyde was determined using human red blood cells according to the method proposed by Jain et al., 2015. Briefly, 80 µL of a 5% erythrocyte/PBS suspension was mixed with 20 µL of cinnamaldehyde at different concentrations and incubated at 37 °C for 1 h. Then, 200 µL of phosphate-buffered saline (PBS; 1.5 mM KH_2_PO_4_, 8.1 mM Na_2_HPO_4_, 136.9 mM NaCl, and 2.6 mM KCl, pH 7.2) was added to stop the hemolysis process, and the samples were centrifuged for 10 min at 1000× *g*. The supernatant was collected, and hemolysis was measured spectrophotometrically (540 nm). The hemolysis percentage was determined as [(Abs_sam_ − Abs_con_)/(Abs_tot_ − Abs_con_) × 100], where Abs_sam_ was the absorbance of the samples, Abs_con_ corresponded to the absorbance of the blank control (without drugs), and Abstot was the absorbance of total hemolysis (replacing the sample solution by an equal volume of Milli-Q water). Study volunteers authorized their participation by signing an informed consent form. The study was conducted in accordance with the Declaration of Helsinki, and the protocol was approved by the Research Ethics Committee at the Federal University of Paraiba under protocol No. 3.430.215 (approved on 2 July 2019) [87].

### 4.9. Antioxidant Activity of Cinnamaldehyde by the DPPH Method

The antioxidant activity of cinnamaldehyde and Trolox (Standard, Sigma Aldrich) was determined based on their radical scavenging effects of stable 2,2-diphenyl-1-picryl-hydrazyl-hydrate (DPPH) free radical by a modified method [88,89]. Solutions with different concentrations of cinnamaldehyde (1.9, 0.75, 0.4, 0.2, and 0.07 mM) were mixed with DPPH (80 µM) and protected from light for 30 min. Then, optical density was measured at 517 nm using a Cecil-Elect Spectrophotometer. Ethanol (1 mL) added of DPPH solution (80 µM, 1 mL) was used as blank. The optical density of the samples was recorded and % inhibition was calculated using the following formula: % Inhibition = [(AbsDPPH 80µM − Abs sample)/Abs DPPH 80µM] × 100. The tests were carried out in triplicate of three independent experiments. IC_50_ values were calculated by linear regression.

## 5. Conclusions

Cinnamaldehyde showed fungicidal activity through a mechanism of action likely related to ergosterol complexation; the best molecular interaction of cinnamaldehyde was with squalene epoxidase; it was non-cytotoxic to keratinocytes and human erythrocytes and showed no antioxidant activity.

## Figures and Tables

**Table 1:** Binding energy (kcal mol^−1^) values of cinnamaldehyde, nystatin and miconazole with the molecular targets 1,3-β-glucan synthase (1EQP), squalene epoxidase (4MAI), δ-14-sterol reductase (4QUV), 14-α-demethylase (5TZ1), and thymidylate synthase (5UIV).

ID	1EQP	4MAI	4QUV	5TZ1	5UIV
Cinnamaldehyde	−61.1458	−70.4951	−59.8535	−51.4852	−66.4852
Miconazole	−135.5710	86.0729	−96.3498	−79.7099	−110.4430
Nystatin	−100.6060	663.2230	21.5918	168.3950	140.0100
Ligand	-	-	−203.0010	−81.7980	−148.3980
RMSD	-	-	0.2048	0.4409	0.2112
Hydrogen Bonds (amount)	Tyr 29 (1) Tyr 255 (1)	Gln 66 (1)	Arg 323 (1) Tyr 407 (1)		Arg 92 (1)
Electrostatic Interactions	-	-	-		
Steric Interactions	Asp 145 (1) Trp 363 (1)	Phe 175 (1) Asn 194 (2)	-	Net 508 (1)	

RMSD: Root Mean Square Deviation.

**Table 2:** Minimum inhibitory concentration (MIC) and Minimum Fungicidal Concentration (MFC) of cinnamaldehyde and nystatin against *Candida* spp. MIC and MFC values are expressed in μM.

	Cinnamaldehyde			Nystatin		
Strain	MIC	MFC	MFC/MIC	MIC	MFC	MFC/MIC
*C. albicans* CBS 562	37.83 µM	37.83 µM	1	8.55 µM	8.55 µM	1
*C. albicans* ATCC 90028	37.83 µM	37.83 µM	1	34.67 µM	34.67 µM	1
*C. krusei* ATCC 6258	18.91 µM	18.91 µM	1	17.33 µM	17.33 µM	1
*C. tropicalis* CBS 94	37.83 µM	37.83 µM	1	17.33 µM	17.33 µM	1
*C. glabrata* ATCC 90030	18.91 µM	18.91 µM	1	8.55 µM	8.55 µM	1
*C. krusei* CBS 573	18.91 µM	18.91 µM	1	34.67 µM	34.67 µM	1

**Table 3:** MIC values of cinnamaldehyde and caspofungin in the absence and presence of sorbitol (0.8 M) against strains of *C. albicans* ATCC 90028 and *C. tropicalis* CBS 94. Values are expressed in μM.

	Cinnamaldehyde					Caspofungin			
Concentration (μM)	*C. albicans* ATCC 90028		*C. tropicalis* CBS 94			*C. albicans* ATCC 90028		*C. tropicalis* CBS 94	
	Without Sorbitol	With Sorbitol	Without Sorbitol	With Sorbitol		Without Sorbitol	With Sorbitol	Without Sorbitol	With Sorbitol
302.6	−	−	−	−	3.6	−	−	−	−
151.3	−	−	−	−	1.8	−	−	−	−
75.6	−	−	−	−	0.9	−	−	−	−
37.8	−	−	−	−	0.4	−	−	−	−
18.9	+	+	+	+	0.2	−	−	−	−
9.4	+	+	+	+	0.1	−	−	−	−
4.7	+	+	+	+	0.05	−	+	−	+
2.3	+	+	+	+	0.02	+	+	+	+

Note: +, fungal growth; −, no fungal growth.

**Table 4:** The effect of exogenous ergosterol (1.008 mM) on the MIC of cinnamaldehyde against *C. albicans* ATCC 90028 and *C. tropicalis* CBS 94. Values are expressed in μM.

	Cinnamaldehyde					Nystatin			
Concentration (μM)	*C. albicans* ATCC 90028		*C. tropicalis* CBS 94			*C. albicans* ATCC90028		*C. tropicalis* CBS 94	
	Without Ergosterol	With Ergosterol	Without Ergosterol	With Ergosterol		Without Ergosterol	With Ergosterol	Without Ergosterol	With Ergosterol
302.6	−	+	−	+	129.5	−	+	−	+
151.3	−	+	−	+	64.7	−	+	−	+
75.6	−	+	−	+	32.3	−	+	−	+
37.8	−	+	−	+	16.1	−	+	−	+
18.9	+	+	+	+	8.0	−	+	−	+
9.4	+	+	+	+	4.0	+	+	−	+
4.7	+	+	+	+	1.9	+	+	+	+
2.3	+	+	+	+	1.0	+	+	+	+

Note: +, fungal growth; −, no fungal growth.

**Table 5:** Antioxidant activity of cinnamaldehyde and Trolox by the DPPH method.

Sample	DPPH Method
	IC_50_ (mM)
Cinnamaldehyde	1.74
Trolox	1.88

IC_50_: Inhibition Concentration of 50%. *n* = 3.

## References

[1] Singh A., Verma R., Murari A., Agrawal A. (2014). Oral Candidiasis: An Overview. J. Oral Maxillofac. Pathol. JOMFP.

[2] Billings M., Dye B.A., Iafolla T., Grisius M., Alevizos I. (2017). Elucidating the Role of Hyposalivation and Autoimmunity in Oral Candidiasis. Oral Dis..

[3] Muadcheingka T., Tantivitayakul P. (2015). Distribution of *Candida albicans* and Non-*albicans Candida* Species in Oral Candidiasis Patients: Correlation between Cell Surface Hydrophobicity and Biofilm Forming Activities. Arch. Oral Biol..

[4] Patil S., Rao R.S., Majumdar B., Anil S. (2015). Clinical Appearance of Oral *Candida* Infection and Therapeutic Strategies. Front. Microbiol..

[5] Manik A., Bahl R. (2017). A Review on Oral Candidal Infection. J. Adv. Med. Dent. Sci. Res..

[6] Junqueira J.C., Vilela S.F.G., Rossoni R.D., Barbosa J.O., Costa A.C.B.P., Rasteiro V., Suleiman J.M.A.H., Jorge A.O.C. (2012). Oral Colonization by Yeasts in HIV-Positive Patients in Brazil. Rev. Inst. Med. Trop. Sao Paulo.

[7] Fox E.P., Nobile C.J., Dietrich L.A., Friedmann T.S. (2013). Candida albicans: Symptoms, Causes and Treatment Options.

[8] Doddanna S.J., Patel S., Sundarrao M.A., Veerabhadrappa R.S. (2013). Antimicrobial Activity of Plant Extracts on *Candida albicans*: An in vitro Study. Indian J. Dent. Res..

[9] Barbosa M.B., Faria M.G.I. (2014). Produtos Naturais Como Nova Alternativa Terapêutica Para o Tratamento de Candidíase Bucal. Rev. Uningá Rev..

[10] Arendrup M.C., Patterson T.F. (2017). Multidrug-Resistant *Candida:* Epidemiology, Molecular Mechanisms, and Treatment. J. Infect. Dis..

[11] Del Valle G.M.M. (2015). *Candida Glabrata:* Un Patógeno Emergente. Biociencias.

[12] El-Houssaini H.H., Elnabawy O.M., Nasser H.A., Elkhatib W.F. (2019). Influence of Subinhibitory Antifungal Concentrations on Extracellular Hydrolases and Biofilm Production by *Candida albicans* Recovered from Egyptian Patients. BMC Infect. Dis..

[13] Perlin D.S., Rautemaa-Richardson R., Alastruey-Izquierdo A. (2017). The Global Problem of Antifungal Resistance: Prevalence, Mechanisms, and Management. Lancet Infect. Dis..

[14] Robbins N., Caplan T., Cowen L.E. (2017). Molecular Evolution of Antifungal Drug Resistance. Annu. Rev. Microbiol..

[15] Simões C.M.O., Schenkel E.P., de Mello J.C.P., Mentz L.A., Petrovick P.R. (2016). Farmacognosia: Do Produto Natural Ao Medicamento.

[16] Khasnavis S., Pahan K. (2012). Sodium Benzoate, a Metabolite of Cinnamon and a Food Additive, Upregulates Neuroprotective Parkinson Disease Protein DJ-1 in Astrocytes and Neurons. J. Neuroimmune Pharmacol..

[17] Malik J., Munjal K., Deshmukh R. (2015). Attenuating Effect of Standardized Lyophilized Cinnamomum Zeylanicum Bark Extract against Streptozotocin-Induced Experimental Dementia of Alzheimer’s Type. J. Basic Clin. Physiol. Pharmacol..

[18] Chung J.-W., Kim J.-J., Kim S.-J. (2011). Antioxidative Effects of Cinnamomi Cortex: A Potential Role of INOS and COX-II. Pharmacogn. Mag..

[19] Mollazadeh H., Hosseinzadeh H. (2016). Cinnamon Effects on Metabolic Syndrome: A Review Based on Its Mechanisms. Iran. J. Basic Med. Sci..

[20] Hamidpour R., Hamidpour M., Hamidpour S., Shahlari M. (2015). Cinnamon from the Selection of Traditional Applications to Its Novel Effects on the Inhibition of Angiogenesis in Cancer Cells and Prevention of Alzheimer’s Disease, and a Series of Functions Such as Antioxidant, Anticholesterol, Antidiabetes, Antibacteri. J. Tradit. Complement. Med..

[21] Connell B.J., Chang S.-Y., Prakash E., Yousfi R., Mohan V., Posch W., Wilflingseder D., Moog C., Kodama E.N., Clayette P. (2016). A Cinnamon-Derived Procyanidin Compound Displays Anti-HIV-1 Activity by Blocking Heparan Sulfate-and Co-Receptor-Binding Sites on Gp120 and Reverses T Cell Exhaustion via Impeding Tim-3 and PD-1 Upregulation. PLoS ONE.

[22] Kim J., Bao T.H.Q., Shin Y.-K., Kim K.-Y. (2018). Antifungal Activity of Magnoflorine against *Candida* Strains. World J. Microbiol. Biotechnol..

[23] Shreaz S., Sheikh R.A., Rimple B., Hashmi A.A., Nikhat M., Khan L.A. (2010). Anticandidal Activity of Cinnamaldehyde, Its Ligand and Ni (II) Complex: Effect of Increase in Ring and Side Chain. Microb. Pathog..

[24] Khan M.S.A., Ahmad I. (2011). Antibiofilm Activity of Certain Phytocompounds and Their Synergy with Fluconazole against *Candida albicans* Biofilms. J. Antimicrob. Chemother..

[25] Khan S.N., Khan S., Iqbal J., Khan R., Khan A.U. (2017). Enhanced Killing and Antibiofilm Activity of Encapsulated Cinnamaldehyde against *Candida albicans*. Front. Microbiol..

[26] Shreaz S., Bhatia R., Khan N., Muralidhar S., Basir S.F., Manzoor N., Khan L.A. (2011). Spice Oil Cinnamaldehyde Exhibits Potent Anticandidal Activity against Fluconazole Resistant Clinical Isolates. Fitoterapia.

[27] Silva D.R., Sardi J.D.C.O., Freires I.A., Silva A.C.B., Rosalen P.L. (2019). In Silico Approaches for Screening Molecular Targets in *Candida albicans:* A Proteomic Insight into Drug Discovery and Development. Eur. J. Pharmacol..

[28] Jovanović M., Obradović R., Pejčić A., Stanišić D., Stošić N., Popović Ž. (2018). The Role of *Candida albicans* on the Development of Stomatitis in Patients Wearing Dentures. Sanamed.

[29] Taudorf E.H., Jemec G.B.E., Hay R.J., Saunte D.M.L. (2019). Cutaneous Candidiasis–an Evidence-based Review of Topical and Systemic Treatments to Inform Clinical Practice. J. Eur. Acad. Dermatology Venereol..

[30] Gow N.A.R., Latge J.-P., Munro C.A. (2017). The Fungal Cell Wall: Structure, Biosynthesis, and Function. Fungal Kingd..

[31] Liu J.-F., Xia J.-J., Nie K.-L., Wang F., Deng L. (2019). Outline of the Biosynthesis and Regulation of Ergosterol in Yeast. World J. Microbiol. Biotechnol..

[32] Choi M., Karunaratne K., Kohen A. (2016). Flavin-Dependent Thymidylate Synthase as a New Antibiotic Target. Molecules.

[33] Pootong A., Norrapong B., Cowawintaweewat S. (2017). Antifungal Activity of Cinnamaldehyde against *Candida albicans*. Southeast Asian J. Trop. Med. Public Health.

[34] Wayne P. (2008). Reference Method for Broth Dilution Antifungal Susceptibility Testing of Yeasts.

[35] Freire J.C.P., Júnior J.K.D.O., Silva D.D.F., Sousa J.P.D., Guerra F.Q.S., de Oliveira Lima E. (2017). Antifungal Activity of Essential Oils against *Candida albicans* Strains Isolated from Users of Dental Prostheses. Evid. Based Complement. Altern. Med..

[36] Scheibler E., Garcia M.C.R., Medina da Silva R., Figueiredo M.A., Salum F.G., Cherubini K. (2017). Use of Nystatin and Chlorhexidine in Oral Medicine: Properties, Indications and Pitfalls with Focus on Geriatric Patients. Gerodontology.

[37] Shreaz S., Bhatia R., Khan N., Muralidhar S., Manzoor N., Khan L.A. (2013). Influences of Cinnamic Aldehydes on H+ Extrusion Activity and Ultrastructure of *Candida*. J. Med. Microbiol..

[38] Shreaz S., Wani W.A., Behbehani J.M., Raja V., Irshad M., Karched M., Ali I., Siddiqi W.A., Hun L.T. (2016). Cinnamaldehyde and Its Derivatives, a Novel Class of Antifungal Agents. Fitoterapia.

[39] Hu L., Wang D., Liu L., Chen J., Xue Y., Shi Z. (2013). Ca^2+^ Efflux Is Involved in Cinnamaldehyde-Induced Growth Inhibition of Phytophthora Capsici. PLoS ONE.

[40] Khan M.S.A., Ahmad I., Cameotra S.S. (2013). Phenyl Aldehyde and Propanoids Exert Multiple Sites of Action towards Cell Membrane and Cell Wall Targeting Ergosterol in *Candida albicans*. Amb Express.

[41] Rajput S.B., Karuppayil S.M. (2013). Small Molecules Inhibit Growth, Viability and Ergosterol Biosynthesis in *Candida albicans*. Springerplus.

[42] Yossa N., Patel J., Millner P., Ravishankar S., Lo Y.M. (2013). Antimicrobial Activity of Plant Essential Oils against *Escherichia Coli* O157: H7 and *Salmonella* on Lettuce. Foodborne Pathog. Dis..

[43] Ferreira G., Rosalen P., Peixoto L., Pérez A., Carlo F., Castellano L., Lima J., Freires I., Lima E., Castro R. (2017). Antibiofilm Activity and Mechanism of Action of the Disinfectant Chloramine T on *Candida* Spp., and Its Toxicity against Human Cells. Molecules.

[44] Taguchi Y., Hasumi Y., Abe S., Nishiyama Y. (2013). The Effect of Cinnamaldehyde on the Growth and the Morphology of *Candida albicans*. Med. Mol. Morphol..

[45] Pakkulnan R., Anutrakunchai C., Kanthawong S., Taweechaisupapong S., Chareonsudjai P., Chareonsudjai S. (2019). Extracellular DNA Facilitates Bacterial Adhesion during *Burkholderia Pseudomallei* Biofilm Formation. PLoS ONE.

[46] Skowron K., Wiktorczyk N., Grudlewska K., Kwiecińska-Piróg J., Wałecka-Zacharska E., Paluszak Z., Gospodarek-Komkowska E. (2019). Drug-Susceptibility, Biofilm-Forming Ability and Biofilm Survival on Stainless Steel of *Listeria* Spp. strains Isolated from Cheese. Int. J. Food Microbiol..

[47] Katchburian E., Arana Chavez V.E. (2012). Histologia e Embriologia Oral: Texto, Atlas, Correlações Clínicas.

[48] García-Salinas S., Elizondo-Castillo H., Arruebo M., Mendoza G., Irusta S. (2018). Evaluation of the Antimicrobial Activity and Cytotoxicity of Different Components of Natural Origin Present in Essential Oils. Molecules.

[49] ISO 10993-5 (1992). Biological Evaluation of Medical Devices—Part 5: Tests for Cytotoxicity: In Vitro Methods.

[50] Yu C., Liu S.-L., Qi M.-H., Zou X. (2014). Cinnamaldehyde/Chemotherapeutic Agents Interaction and Drug-Metabolizing Genes in Colorectal Cancer. Mol. Med. Rep..

[51] Divkovic M., Pease C.K., Gerberick G.F., Basketter D.A. (2005). Hapten–Protein Binding: From Theory to Practical Application in the in vitro Prediction of Skin Sensitization. Contact Dermat..

[52] Suwalsky M., Vargas P., Avello M., Villena F., Sotomayor C.P. (2008). Human Erythrocytes Are Affected *in vitro* by Flavonoids of Aristotelia Chilensis (Maqui) Leaves. Int. J. Pharm..

[53] Dobrovolskaia M.A., McNeil S.E. (2013). Understanding the Correlation between in vitro and in Vivo Immunotoxicity Tests for Nanomedicines. J. Control. Release.

[54] Farag M.R., Alagawany M. (2018). Erythrocytes as a Biological Model for Screening of Xenobiotics Toxicity. Chem. Biol. Interact..

[55] Scherer R., Wagner R., Duarte M.C.T., Godoy H.T. (2009). Composição e Atividades Antioxidante e Antimicrobiana Dos Óleos Essenciais de Cravo-Da-Índia, Citronela e Palmarosa. Rev. Bras. Plantas Med..

[56] Release H. (2002). 7.5 for Windows, Molecular Modeling System.

[57] De Campos L.V.B., Correia J.C.G., Carauta A.N.M. (2017). Estudo Da Interação Do Trietoxisilano Com o Ácido Linoléico Como Hidrofugante Em Rochas Ornamentais via Modelagem Molecular.

[58] Moreira M.P. (2017). Novos Polímeros a Base de Ácido Glicerofosfórico/Beta-Ciclodextrina Reticulado Com Ligações Uretânicas: Preparação e Incorporação de Ciprofloxacina.

[59] Altê M.A. (2017). Estudo Estrutural e Planejamento de Novos Inibidores Para a Enzima Prefenato Desidratase de Mycobacterium Tuberculosis.

[60] Barros R.P.C. (2017). Triagem Virtual de Metabólitos Secundários Com Potencial Atividade Antimicrobiana Do Gênero Solanum e Estudo Fitoquimico de Solanum Capsicoides All.

[61] Santana C.B. (2017). Composição Química, Atividade Antimicrobiana, Inseticida e Antioxidante Do Óleo Essencial e Extratos de Myrcia Oblongata DC.

[62] Hargrove T.Y., Friggeri L., Wawrzak Z., Qi A., Hoekstra W.J., Schotzinger R.J., York J.D., Guengerich F.P., Lepesheva G.I. (2017). Structural Analyses of *Candida albicans* Sterol 14α-Demethylase Complexed with Azole Drugs Address the Molecular Basis of Azole-Mediated Inhibition of Fungal Sterol Biosynthesis. J. Biol. Chem..

[63] Li X., Roberti R., Blobel G. (2015). Structure of an Integral Membrane Sterol Reductase from *Methylomicrobium alcaliphilum*. Nature.

[64] Cutfield J.F., Sullivan P.A., Cutfield S.M. (2000). Minor Structural Consequences of Alternative CUG Codon Usage (Ser for Leu) in *Candida albicans* Exoglucanase. Protein Eng..

[65] Sinha K., Rule G.S. (2017). The Structure of Thymidylate Kinase from *Candida albicans* Reveals a Unique Structural Element. Biochemistry.

[66] Hemsworth G.R., Henrissat B., Davies G.J., Walton P.H. (2014). Discovery and Characterization of a New Family of Lytic Polysaccharide Monooxygenases. Nat. Chem. Biol..

[67] Ali S.E., Chehri K., Karimi N., Karimi I. (2017). Computational Approaches to the *in vitro* Antibacterial Activity of *Allium airtifolium* Boiss against Gentamicin-Resistant *Escherichia Coli:* Focus on Ribosome Recycling Factor. Silico Pharmacol..

[68] Varma P.B.S., Adimulam Y.B., Subrahmanyam K. (2017). *In Silico* Virtual Screening of PubChem Compounds against Phosphotransacetylase, a Putative Drug Target for *Staphylococcus aureus*. Int. J. Comput. Biol. Drug Des..

[69] Wu Y.-Y., Zhang T.-Y., Zhang M.-Y., Cheng J., Zhang Y.-X. (2018). An Endophytic Fungi of *Ginkgo Biloba* L. Produces Antimicrobial Metabolites as Potential Inhibitors of FtsZ of *Staphylococcus aureus*. Fitoterapia.

[70] Loo J.S.E., Emtage A.L., Ng K.W., Yong A.S.J., Doughty S.W. (2018). Assessing GPCR Homology Models Constructed from Templates of Various Transmembrane Sequence Identities: Binding Mode Prediction and Docking Enrichment. J. Mol. Graph. Model..

[71] Ounthaisong U., Tangyuenyongwatana P. (2017). Cross-Docking Study of Flavonoids against Tyrosinase Enzymes Using PyRx 0.8 Virtual Screening Tool. TJPS.

[72] Wang T., Yang Z., Zhang Y., Yan W., Wang F., He L., Zhou Y., Chen L. (2017). Discovery of Novel CDK8 Inhibitors Using Multiple Crystal Structures in Docking-Based Virtual Screening. Eur. J. Med. Chem..

[73] Yanuar A., Pratiwi I., Syahdi R.R. (2018). In Silico Activity Analysis of Saponins and 2, 5-Piperazinedione from Marine Organism against Murine Double Minute-2 Inhibitor and Procaspase-3 Activator. J. Young Pharm..

[74] Rasooli I., Abyaneh M.R. (2004). Inhibitory Effects of Thyme Oils on Growth and Aflatoxin Production by Aspergillus Parasiticus. Food Control.

[75] Siddiqui Z.N., Farooq F., Musthafa T.N.M., Ahmad A., Khan A.U. (2013). Synthesis, Characterization and Antimicrobial Evaluation of Novel Halopyrazole Derivatives. J. Saudi Chem. Soc..

[76] Frost D.J., Brandt K.I.M.D., Cugier D., Goldman R. (1995). A Whole-Cell *Candida albicans* Assay for the Detection of Inhibitors towards Fungal Cell Wall Synthesis and Assembly. J. Antibiot..

[77] Leite M.C.A., Bezerra A.P.D.B., Sousa J.P.D., Guerra F.Q.S., Lima E.D.O. (2014). Evaluation of Antifungal Activity and Mechanism of Action of Citral against *Candida albicans*. Evid. Based Complement. Altern. Med..

[78] De Almeida Freires I., Murata R.M., Furletti V.F., Sartoratto A., de Alencar S.M., Figueira G.M., de Oliveira Rodrigues J.A., Duarte M.C.T., Rosalen P.L. (2014). *Coriandrum sativum* L.(Coriander) Essential Oil: Antifungal Activity and Mode of Action on *Candida* Spp., and Molecular Targets Affected in Human Whole-Genome Expression. PLoS ONE.

[79] Lima I.O., Pereira F.D., Oliveira W.A., Lima E.D., Menezes E.A., Cunha F.A., Diniz M.D. (2013). Antifungal Activity and Mode of Action of Carvacrol against *Candida albicans* Strains. J. Essent. Oil Res..

[80] Hao B., Cheng S., Clancy C.J., Nguyen M.H. (2013). Caspofungin Kills *Candida albicans* by Causing Both Cellular Apoptosis and Necrosis. Antimicrob. Agents Chemother..

[81] Letscher-Bru V., Herbrecht R. (2003). Caspofungin: The First Representative of a New Antifungal Class. J. Antimicrob. Chemother..

[82] Pierce C.G., Srinivasan A., Uppuluri P., Ramasubramanian A.K., Lopez-Ribot J.L. (2013). Antifungal Therapy with an Emphasis on Biofilms. Curr. Opin. Pharmacol..

[83] Ellepola A.N.B., Samaranayake L.P. (2014). Impact of Brief and Sequential Exposure to Nystatin on the Germ Tube Formation and Cell Surface Hydrophobicity of Oral *Candida albicans* Isolates from Human Immunodeficiency Virus-Infected Patients. Med. Princ. Pract..

[84] Alves L.A., Freires I.D., Pereira T.M., Souza A.D., Lima E.D., Castro R.D. (2013). Effect of Schinus Terebinthifolius on *Candida albicans* Growth Kinetics, Cell Wall Formation and Micromorphology. Acta Odontol. Scand..

[85] Ellepola K., Liu Y., Cao T., Koo H., Seneviratne C.J. (2017). Bacterial GtfB Augments *Candida albicans* Accumulation in Cross-Kingdom Biofilms. J. Dent. Res..

[86] Denizot F., Lang R. (1986). Rapid Colorimetric Assay for Cell Growth and Survival: Modifications to the Tetrazolium Dye Procedure Giving Improved Sensitivity and Reliability. J. Immunol. Methods.

[87] Jain K., Verma A.K., Mishra P.R., Jain N.K. (2015). Surface-Engineered Dendrimeric Nanoconjugates for Macrophage-Targeted Delivery of Amphotericin B: Formulation Development and *in vitro* and in Vivo Evaluation. Antimicrob. Agents Chemother..

[88] Brand-Williams W., Cuvelier M.-E., Berset C. (1995). Use of a Free Radical Method to Evaluate Antioxidant Activity. LWT Food Sci. Technol..

[89] Kim D.-O., Lee K.W., Lee H.J., Lee C.Y. (2002). Vitamin C Equivalent Antioxidant Capacity (VCEAC) of Phenolic Phytochemicals. J. Agric. Food Chem..

